# Annotations

**Published:** 1895-12-21

**Authors:** 


### ANNOTATIONS.

### The Poisoner, the Doctor, and the Nurse.

Poisoning will soon become a lost art. In what has for some time been known in West Yorkshire as the "Huddersfield poisoning case, Mrs. Rowland Robinson essayed to put an end to her husband's life by poisoning him with white precipitate, scientifically known as the ammonio-chloride of mercury. Dose after dose failed, and at length the doctor was called in. Even after his attendance had commenced, the infatuated woman, strong in her faith in the doctor's stupidity, persisted in her administration of the poison. Very soon the doctor noted the physiological effects of large doses of mercury; very soon his suspicions of foul play were aroused; in vain he asked for the vomit and the dejecta to be kept for him; the poisoner knew her guilt too well for that. Not to be baffled, the doctor insisted upon a trained nurse. Kathleen Campion was her name. Then the poisoner held her hand for awhile. But there was no happiness, she thought, in her mind or her circumstances until her husband was "put away." Again, therefore, she drops the poison into his milk; some of it is administered, and produces vomiting and diarrhœa as before. Then was the nurse's opportunity. With admirable promptitude she secured portions of the vomit, the motions, and the poisoned food. In all three the white precipitate was found, and in quantities sufficient to kill half-a-dozen husbands. That was the last of Mrs. Robinson's attempts. The law took charge of her. The adulterous lover was brought to light; so also was a will made by the victim in his wife's favour, and a policy of insurance on his life; with these, and the presence of the poison in food, vomit, and motions, together with the evidence of the doctor and the nurse, conviction was certain. In less than five minutes the Yorkshire jury brought in a verdict of guilty, and a stern judge had condemned the would-be murderess to twenty years' penal servitude. The case is a striking object lesson of the certainty of science, and will undoubtedly impress the imagination of would-be murderers with the difficulty and practical impossibility of safely carrying out their nefarious schemes—at least, by the methods of the crude and common poisoner.

### Drunk or Dying.

A case which occurred at St. Bartholomew's Hospital last week, in which fracture of the skull was overlooked, apparently in consequence of the accompanying head symptoms being attributed to drink, brings into prominence once more a point to which we have before alluded, viz., the great importance of some systematic routine being adopted at our hospitals by which such lamentable occurrences should be rendered, if not impossible, at any rate far less frequent than they are at present. In regard to this case we think some further inquiry should be made—not so much as to the action of this or that house surgeon, but as to the system by which the officers of the hospital are supposed to be governed under similar circumstances. So far as we can judge from the report with which we are furnished the patient was brought to the hospital with a history of having fallen backwards on to his head while standing on the shafts of a van. He was suffering from a scalp wound; on his way he had staggered and spoken strangely, and he was noticed by the dresser to be incoherent and to hesitate in giving his name and address. His wound was attended to by the dresser, and he was seen by the house surgeon, a qualified man, by whom he was discharged. All was done in proper form, but a grave error of diagnosis was committed. His mental condition was attributed to drink, and on the dresser expressing the opinion that he was "far from sober" he was taken into custody. When placed in the dock he suddenly shrieked and fell down paralysed, and on his death it was discovered that not only had he a fracture of the skull, but that some of his ribs also were broken. Now this is a very distressing and serious case. We are far from saying that anything which might have been done would have saved the man, but that there are cases of similar character in which more careful treatment might make all the difference between life and death we are convinced, and we are sure that in this matter the leading teachers will be with us. The difficulty of being sure as to the meaning of the earlier and slighter degrees of drunkenness is so proverbial, and the doubt which hangs over every case of head injury until all cerebral symptoms have passed away is so well-known that we find it difficult to believe that any young man, fresh from the mill of the lecture-room and the examination hall, would run the risk of all the obloquy which he must know would fall upon him in case of a mistake unless his action in not admitting the patient to the wards were influenced by some system or tradition which runs counter to the knowledge which is now common to all. That a horrible blunder was made there can be no doubt, and it is not the first. What we are anxious about is how far the system now current in that hospital in regard to the detention of doubtful cases will help to ensure or to prevent the recurrence of such catastrophes.

### The Chelsea Hospital.

We have received a lengthy report of the quarrel now pending between the Board of the Chelsea Hospital for Women and Mr. O'Callaghan, who, it will be remembered, was appointed surgeon to this hospital when the staff was reorganized a year ago. We will not say anything about the personal matters which have been introduced, but it is difficult to avoid a feeling that the maintenance of the old leaven in the new administration has left unchanged some of the elements of friction which existed before. As regards the particular point which seems to have brought smouldering jealousies to a climax, we confess that, so far as Mr. O'Callaghan endeavoured to personally undertake the after-treatment of his more severe operations, offering to keep within call for the purpose, he was only following the teaching of modern surgery in thus limiting the chances of wound infection, especially in view of the attention which had been drawn to the prevalence of septic disorders under the old régime by the Committee of Inquiry which investigated the affairs of the hospital last year.

